# Rho GTPase Signaling in Platelet Regulation and Implication for Antiplatelet Therapies

**DOI:** 10.3390/ijms24032519

**Published:** 2023-01-28

**Authors:** Akhila Dandamudi, Huzoor Akbar, Jose Cancelas, Yi Zheng

**Affiliations:** 1 Division of Experimental Hematology and Cancer Biology, Cincinnati Children’s Hospital Medical Center, 3333 Burnet Avenue, Cincinnati, OH 45229, USA; 2 Department of Pathology, University of Cincinnati Graduate School, Cincinnati, OH 45267, USA; 3 Department of Biomedical Sciences, Heritage College of Osteopathic Medicine, Ohio University, Athens, OH 45701, USA; 4 Hoxworth Blood Center, College of Medicine, University of Cincinnati, Cincinnati, OH 45229, USA

**Keywords:** platelet activation, thrombosis, cell signaling, rational targeting, small molecule inhibitors

## Abstract

Platelets play a vital role in regulating hemostasis and thrombosis. Rho GTPases are well known as molecular switches that control various cellular functions via a balanced GTP-binding/GTP-hydrolysis cycle and signaling cascade through downstream effectors. In platelets, Rho GTPases function as critical regulators by mediating signal transduction that drives platelet activation and aggregation. Mostly by gene targeting and pharmacological inhibition approaches, Rho GTPase family members RhoA, Rac1, and Cdc42 have been shown to be indispensable in regulating the actin cytoskeleton dynamics in platelets, affecting platelet shape change, spreading, secretion, and aggregation, leading to thrombus formation. Additionally, studies of Rho GTPase function using platelets as a non-transformed model due to their anucleated nature have revealed valuable information on cell signaling principles. This review provides an updated summary of recent advances in Rho GTPase signaling in platelet regulation. We also highlight pharmacological approaches that effectively inhibited platelet activation to explore their possible development into future antiplatelet therapies.

## 1. Introduction

**Figure 1:**
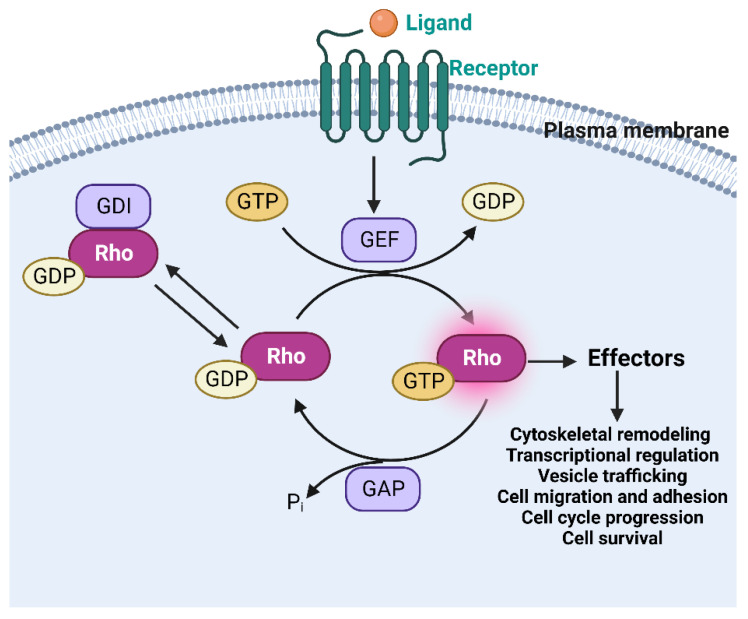
The Rho GTPase signaling cycle. Rho GTPases typically cycle between the active GTP-bound active form and the GDP-bound inactive form. This GTP-binding/GTP-hydrolysis cycle is primarily regulated by three classes of proteins: GEFs, GAPs, and GDIs. GEFs catalyze the exchange of GDP to GTP, activating the Rho GTPase, while the GAPs inactivate the RhoGTPase by hydrolyzing the GTP. The GDIs sequester and extract the Rho GTPases from the membrane to prevent the interactions between Rho and GEFs, GAPs, and downstream effectors. The activated Rho GTPase turns on effectors to transduce signals leading to platelet cell functional changes.

Rho family GTPases are small (~ 21 kDa) signaling G proteins of the Ras superfamily. Like most conventional GTPases, Rho GTPases function as molecular switches by cycling between the inactive GDP-bound form and the active GTP-bound form by a mechanism regulated by three classes of regulators: Guanine nucleotide exchange factors (GEFs), GTPase activating proteins (GAPs), and GDP dissociation inhibitor (GDI). GEFs catalyze the nucleotide exchange from GDP to GTP, leading to Rho GTPase activation, while the GAPs stimulate the hydrolysis of bound GTP to GDP, resulting in Rho GTPase inactivation. GDIs extract the inactive Rho GTPases from the membranes to prevent the interaction between Rho and GEFs, GAPs, and downstream signaling mediators called “effectors” [1,2,3]. Upon activation, Rho GTPases turn on effectors to regulate a variety of cellular processes, such as cytoskeletal remodeling, transcriptional regulation, vesicle trafficking, migration, adhesion, cell cycle progression, and cell survival (Figure 1) [4,5,6,7].

Platelets play a fundamental role in hemostasis and thrombosis. Under normal physiological conditions, platelets circulate freely in the vasculature. However, upon vascular injury or rupture of an atherosclerotic plaque, they rapidly adhere to the subendothelial matrix and become activated by undergoing cytoskeletal rearrangement, shape change, granular secretion, aggregation, and thrombus formation [8]. Platelets are independent of nuclear events, making them a useful non-transformed model to study Rho GTPase signaling. Among the Rho GTPase members, RhoA, Cdc42, and Rac1 have the best established roles as regulators of platelet function and are well characterized in terms of cellular processes and signaling transduction [9,10,11,12,13,14,15,16,17,18,19,20,21,22,23,24,25,26]. This review provides a summary of recent studies of Rho GTPase signaling in platelet hemostasis and thrombosis and the implications of developing new approaches targeting individual Rho GTPases in potential antiplatelet therapies.

## 2. Platelet Structure

The first accurate and convincing description of platelets was first published in 1865 by Max Schultz [27]. However, Giulio Bizzozzero is regarded as the “discoverer of the platelet” since he was the first person to observe the platelets microscopically and their contribution to promoting thrombosis in vivo and blood coagulation in vitro in the year 1882 [28]. He described platelet function in flowing conditions and the relationship between platelet activation, adhesion, aggregation, and fibrin formation and deposition [29]. Platelets or thrombocytes are the smallest disc-shaped anucleate fragment of a bone marrow megakaryocyte, the only polyploid hematopoietic cell in mammals. Platelets average 2–5 μm in diameter, 0.5 μm in thickness, and have a mean cell volume of 6 to 10 femtoliters [30]. Human platelets have a lifespan of 7 to 10 days, with the average platelet count ranging from 150,000 to 350,000 platelets per microliter of blood [31].

Due to the role of platelets in hemostasis and thrombosis, it is vital to understand the platelet structure and structural physiology. Platelets have four main components: the peripheral zone, the sol-gel zone, the organelle zone, and the platelet membrane system [32]. The peripheral zone comprises the platelet plasma membrane (lipid bilayer), which has a thicker exterior coat referred to as the glycocalyx, the open canalicular system (OCS), and the sub-membrane filaments. The glycocalyx plays a vital role in the acceleration of clotting [33]. The OCS contributes to an increase in the surface area in platelet spreading [34]. The sub-membrane filaments regulate the shape change and the translocation of receptors and particles over the exterior surface of the cell [35]. The sol-gel zone is a transparent but viscous matrix containing secretory organelles, glycogen, smooth and clathrin-coated vesicles, microtubules, and microfilaments [36]. The microtubules function as cytoskeletal support systems, and the actomyosin filament system is involved in platelet shape change and clot retraction [37]. The glycogen granules could serve as a reservoir for energy production, and the smooth and coated vesicles fill the cytoplasm close to the Golgi complexes [38]. The organelle zone has α-granules, dense granules, and lysosomes in addition to mitochondria, glycosomes [39], tubular inclusions [40], electron-dense chains, and clusters [41]. The α-granules are the most abundant and largest secretory organelles containing von Willebrand factor (vWF), coagulation factor V, thrombospondin, fibrinogen, and p-selectin, which are responsible for maintaining normal platelet activity [42]. The dense bodies are fewer in number and smaller than the α-granules and have high morphological activity [43]. The dense bodies are rich in adenosine triphosphate (ATP), adenosine diphosphate (ADP), serotonin, pyrophosphate, calcium, and magnesium. The role of lysosomes, electron-dense chains and clusters, and glycosomes in platelet function is unknown. Platelet mitochondria and the dense tubular system (DTS) are responsible for the energy requirements during platelet activation and granule content release [44]. Finally, the platelet membrane system comprises the Golgi zones, the surface-connected OCS, DTS, membrane complexes, and the rough endoplasmic reticulum. The Golgi zones are confined to the megakaryocyte during granulopoiesis and almost completely disappear before proplatelets develop. The surface-connected OCS is a surface membrane connected to the platelet surface membrane, which is responsible for the increase in platelet surface area and serves as a conduit for the discharge of products stored in secretory organelles during platelet release reaction [45]. The DTS is formed from the residual channels of the smooth and rough endoplasmic reticulum, which has the calcium-binding sites and enzymes involved in prostaglandin synthesis [46]. The channels of the OCS and the elements of the DTS form the membrane complexes, whose precise function is unclear [47]. The rough endoplasmic reticulum is the rarest of the membrane systems and usually disappears before the proplatelets are formed. The final stage of the rough endoplasmic reticulum is when the smooth endoplasmic reticulum makes up the DTS [32].

These complex structures endow platelets with their unique function through evolution in mediating diverse physiologic and pathologic responses in hemostasis.

## 3. Role of Platelets in Hemostasis and Thrombosis

Hemostasis is the clot formation process that stops bleeding from a blood vessel, while thrombosis is the formation of clots within the blood vessels that limits the blood flow [8,27]. When vascular endothelium is ruptured due to injuries or trauma to the blood vessels, vascular spasms promote vasoconstriction [48]. Under high shear, platelets adhere to the collagen bound to vWF present on the exposed extracellular matrix (ECM) via the glycoprotein receptor, GPIb-V-IX complex, on the platelet surface membrane [49]. This GPIb-V-IX interaction enables platelet tethering to the damaged vessel wall and slows platelets, allowing the interaction between GPVI and collagen [50]. GPVI interaction with collagen activates the downstream signaling events by facilitating platelet activation and stable adhesion via integrin α2β1 [51]. At the same time, platelets release a variety of cytoplasmic granules containing ADP, ATP, thrombin, serotonin, and thromboxane A2 (TxA2) [52]. These mediators trigger a series of signaling pathways via their respective G protein-coupled receptors (GPCRs) that amplify platelet activation and aggregation. For example, ADP acts via two GPCRs in humans: 1) P2Y1 receptor couples to Gq/11 to initiate platelet shape change and mobilization of the internal calcium stores resulting in ADP-induced aggregation. 2) P2Y12 receptor acts via Gi and couples with the adenylyl cyclase inhibition resulting in a complete aggregation response to ADP and stabilizing aggregates [53]. ATP acts as an antagonist on the P2Y receptors and initiates platelet activation by mediating the ionic fluxes via the ligand-gated ion channel receptor, P2X1 [54]. Thrombin is a potent platelet agonist which activates protease-activated receptors (PARs) such as PAR1 and PAR4 in humans [55]. Serotonin is an indolamine that binds to the platelet serotonin receptor (5-HT2A) and has vasoconstrictive and aggregating properties [56,57]. TxA2 is an unstable arachidonate metabolite that induces platelet aggregation and is a known vasoconstrictor of vascular and respiratory smooth muscles [58]. Platelets undergo activation and spreading, which activates the integrin αIIbβ3, resulting in stable platelet adhesion, platelet aggregation, and thrombus formation [59]. The agonist-dependent intracellular signals regulate the activation of integrins from a “low affinity” state to a “high affinity” state, which results in increased affinity to its extracellular ligands such as fibronectin, vWF, fibrinogen, and collagen (“inside-out” signaling) [60]. The binding of integrin αIIbβ3 to the ligand also initiates a series of intracellular signaling events resulting in cytoskeletal reorganization, granule secretion, stable adhesion, and clot retraction (“outside-in” signaling) [61]. Activated platelets recruit additional platelets to the injured vessel wall, forming a platelet plug (primary hemostasis). However, more profound tissue damage results in tissue factor (TF) release, which eventually activates the coagulation cascade [62].

The coagulation cascade consisting of the intrinsic (contact activation pathway) and extrinsic (tissue factor) pathway is triggered by a series of reactions mediated by coagulation mediators. Platelets act as the catalytic site for the formation, release, and activation of the coagulation mediators and generation of thrombin, which converts fibrinogen to fibrin [63]. In the final step of the coagulation cascade, fibrin enmeshes the plasma, platelets, and blood cells, building a stable clot. Tertiary hemostasis involves the lysis of the fibrin clot resulting in clot resolution and restoration of blood flow in the damaged or obstructed blood vessels [63].

Under certain pathological conditions, misleading, exaggerated hemostatic response results in the formation of a blood clot inside a blood vessel, eventually leading to thrombosis. These blood clots sometimes break off and are carried through the bloodstream, lodging in the vessels known as embolus, resulting in thromboembolism [64]. Thromboembolic conditions are the leading cause of mortality worldwide, and they are divided into arterial and venous thrombotic conditions [65]. The location and composition of the thrombus are used to classify thrombosis as arterial or venous thrombosis. Arterial thrombosis occurs due to clot formation during an atherosclerotic rupture resulting in platelet aggregation, thrombus formation, vessel occlusion, and possible ischemic heart disease or ischemic stroke [66]. Arterial thrombi are formed at relatively high shear rates (~102–105 s^−1^) and have significant amounts of platelet aggregates and fibrin but fewer red blood cells (RBCs), resulting in the name “white thrombi.” Antiplatelet therapy is the preferred method for arterial thrombosis since it is generally regarded as a platelet-related disease [67]. Venous thrombosis develops within a vein and occurs in the case of endothelial injury, stasis, or hypercoagulability, resulting in deep-vein thrombosis (DVT) or pulmonary embolism (PE) [68]. Venous thrombi are formed under low shear rates (10–100 s^−1^), mainly composed of RBCs and fibrin, and fewer platelets, resulting in the name “red thrombi” [66]. Venous thrombosis is generally regarded as a coagulation-related disease making anticoagulant therapy the preferred treatment method [69].

## 4. Rho GTPases in Platelets

A wealth of studies have implicated Rho GTPase family members like RhoA, Rac1, and Cdc42 as key regulators in platelet shape change, aggregation, secretion, and thrombus formation due to their role in cytoskeletal rearrangements [9,10,11,12,13,14,15,16,17,18,19,20,21,22,23,24,25,26]. Studies of Rho GTPases have contributed to numerous discoveries about platelet functions in hemostasis and thrombosis [10]. Due to the conserved actin pathways in platelets, they are also valuable tools for studying Rho GTPase function [11]. Experiments using substrate protein for botulinum C3 ADP-ribosyltransferase (C3 exoenzyme), a Rho inhibitor, helped identify and define the role of Rho protein family members in several cell types, including platelets [9,12]. RhoA is well known for platelet shape change upon activation and thrombus stability due to its role in mediating actin contractility [13,14,15,16]. Rac1 is vital for platelet lamellipodia formation, stability of platelet aggregates under flow, secretion, and platelet aggregation [17,18,19]. The role of Cdc42 in platelet function is complex, but Cdc42 activation is associated with platelet filopodia formation, secretion, and platelet aggregation [20,21,22]. Another relatively less studied Rho GTPase, RhoG, is involved in GPVI/FcRγ-mediated platelet activation, granule secretion, and is critical for thrombus formation in vivo [70,71,72]. Although each Rho GTPase has a specialized function in platelet activation, growing evidence indicates that complex interdependent crosstalks between Rho GTPases, their regulators, and effectors are at play [10,16,23,24,25,26].

### 4.1. Role of RhoA in Platelet Function

**Figure 2:**
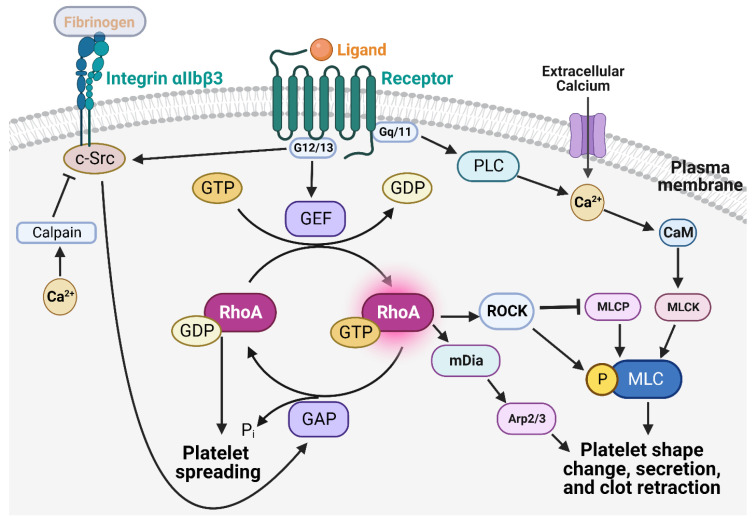
Role of RhoA in platelet activation and aggregation. RhoA works downstream of multiple G-protein coupled receptors and integrins to mediate platelet shape change, spreading, secretion, and clot retraction. Gα13 activates RhoGEF promoting RhoA-GTP formation, which eventually phosphorylates its downstream effector Myosin light chain (MLC), resulting in shape change and secretion. Gα13 also interacts with integrin αIIbβ3 to activate Src family kinases, which activate RhoGAP, which leads to RhoA inhibition, allowing platelets to spread. Calcium released during the initial stages of platelet activation causes calpain to cleave integrin β3 inhibiting c-Src activation to promote contractility and clot retraction. In addition to Gα13, Gq/11 also contributes to platelet activation via calcium/calmodulin-mediated MLC kinase activation.

RhoA is a crucial regulator of platelet function in hemostasis and thrombosis in vivo and is likely the most highly expressed Rho isoform in human platelets, with an estimated copy number per platelet of ~31,300 [73]. In platelets, RhoA works downstream of a number of GPCR pathways to promote platelet shape change, granule secretion, spreading, and clot retraction via its effector molecules such as Rho-associated, coiled-coil–containing protein kinase (ROCK) and mammalian diaphanous homolog (mDia). RhoA nucleates new actin filaments via mDia1 and stabilizes actin filaments via ROCK and LIM-kinase [70]. The RhoA-ROCK-MLC axis is cyclically targeted via GPCR pathways and integrin outside-in signaling pathways to regulate platelet activation dynamically. Upon agonist stimulation, GTP-bound Gα13 activates RhoGEF, which promotes RhoA-GTP formation, which binds to and activates ROCK. The activated ROCK phosphorylates and inactivates MLC phosphatase resulting in an increase of the phosphorylated MLC and actomyosin contractions that promotes platelet shape change and secretion [74]. Next, Gα13 interacts with the cytosolic domain of integrin αIIbβ3 to activate Src family kinases (SFK). SFK activates RhoGAP, which leads to RhoA inhibition to allow platelet spreading [75,76]. Subsequently, calcium released from the intracellular stores during the initial stages of platelet activation causes the calpain to cleave integrin β3, which inhibits c-Src activation to stabilize thrombi under shear and promotes contractility and clot retraction [14,77]. In addition to the Gα13-RhoA-ROCK pathway, Gq/11 also contributes to an increase of the phosphorylated MLC by activating phospholipase Cβ (PLC), which results in calcium/calmodulin-mediated stimulation of MLC kinase (Figure 2) [78].

There have been two different strategies in the generation of the RhoA knockout model in mice, which might have contributed to inconsistent conclusions regarding the role of RhoA in platelet function. The two strategies employed to eliminate RhoA expression in mice are by using an inducible deletion of RhoA in the hematopoietic cells by polyinosinic acid-polycytidylic acid induction (Mx-Cre RhoA^fl/fl^) or by a Cre-recombinase model that targets RhoA in megakaryocytes and platelets directly via the platelet factor 4 (Pf4) promoter during megakaryocyte maturation (Pf4-Cre RhoA^fl/fl^) [15,16]. Experiments using megakaryocytes/platelet-specific RhoA-deficient mice (Pf4-Cre RhoA^fl/fl^) showed that RhoA is involved in different cellular responses downstream of Gα13 and Gq/11-coupled agonist receptors and activation of integrin αIIbβ3, but RhoA is not required for platelets to spread over the surface of fibrinogen, an integrin αIIbβ3 substrate [15]. These platelets have impaired shape change, granule secretion, clot retraction, and mild aggregation defects downstream of Gα13 and Gq/11-activation. They also showed that these in vitro observations translated into in vivo functions where the RhoA-deficient mice showed reduced thrombus formation, had prolonged tail-bleeding times, and partial protection from ischemic stroke [15]. In another study, gene targeting of RhoA (Mx-Cre RhoA^fl/fl^) or pharmacological inhibition of RhoA in platelets using Rho specific inhibitor, Rhosin, a small molecule RhoA inhibitor, showed that RhoA facilitates the generation of reactive oxygen species (ROS), phosphorylation of MLC and platelet shape change in addition to platelet spreading on immobilized fibrinogen [16]. The discrepancies about the involvement of RhoA in integrin αIIbβ3-dependent platelet spreading could be attributed to the different knockout strategies employed to eliminate RhoA expression due to a difference in the density of fibrinogen and/or the duration of time platelets were exposed to fibrinogen [15,16]. Treating platelets with the C3 enzyme, which mainly inhibits RhoA, or treating them with the ROCK inhibitor Y-27632 blocked the shape change and actin polymerization in Gq/11 deficient systems in which Gα12/Gα13 are active [13]. Thus, RhoA inhibition in platelets by using RhoA deficient mice or by pharmacological targeting showed that RhoA plays a fundamental role in integrin activation, platelet spreading, granule secretion, and clot retraction [15,16].

### 4.2. Role of Rac1 in Platelet Function

Experiments attempting to identify the Rac isoforms present in platelets showed that out of the Rac1, Rac2, and Rac3 isoforms, Rac1 is predominantly expressed in human platelets (estimated copy number per platelet ~ 32,900) and murine platelets [17,73]. Constitutively active and dominant negative mutants of Rac have been used to establish the role of Rac1 in the production of lamellipodia and membrane ruffles [6,79]. Studies using Rac1 deficient mice showed that Rac1 is essential for platelet lamellipodia but not filopodia formation, actin polymerization, and aggregate integrity, leading to thrombus formation under dynamic flow conditions both in vitro and in vivo [17,18,79,80]. Diverse agonists activate Rac1 via their specific receptors and downstream signaling cascades, stimulating PLC and calcium mobilization [81]. The cytosolic calcium then activates gelsolin resulting in calcium-dependent actin filament-uncapping and severing activity and transient association with their barbed ends [82]. Rac also stimulates actin polymerization through the SCAR (suppressor of cyclic AMP receptor)/WAVE (Wiskott–Aldrich syndrome protein (WASP) family verprolin-homologous protein) family which activates the Arp2/3 complex responsible for nucleating the actin-barbed ends by forming branches on actin filaments near the protruding edges of the cell [80,83,84]. The gelsolin and Rac pathways act in tandem through uncapping, severing, and nucleation to cause rapid actin reorganization of new filaments at the leading edge of lamellipodia [80,85]. Activated Rac1 has also been implicated in the stimulation of PLC-γ2 and phosphoinositide-3 kinase (PI3K) [86,87]. PI3K can also be activated via the agonist stimulation of GPCRs which ultimately activates Rac1 [88]. Rac1 is a key regulator of platelet activation due to the bidirectional positive feedback loops for the activation of Rac1 by PLC-γ2 and PI3K and vice-versa. Upon activation, Rac and Cdc42 interact with their downstream effector, p21-activated kinases (PAKs), and PAKs play a key role in the cytoskeletal organization, including lamellipodia formation [89].

Experiments using a dual approach of gene targeting (Mx-Cre Rac1^fl/fl^ or Pf4-Cre Rac1^fl/fl^) and pharmacologic inhibition (NSC23766, a rationally designed specific small molecule Rac1 inhibitor) showed that Rac1 GTPase is involved in the regulation of platelet secretion which in part results in diminished platelet aggregation as well as prolonged bleeding time in vivo [18,90]. Rac1 is also implicated in platelet activation via the CRP (collagen-related peptide)-GPVI mediated ROS generation [91]. Another Rac1 knockout study showed that Rac1 is essential for immunoreceptor tyrosine activation motif (ITAM) dependent PLC-γ2 activation and thrombus formation in vitro and in vivo [92]. Rac1 inhibition in platelets by using Rac1 deficient mice or pharmacological targeting showed that Rac1 is a crucial player in lamellipodia formation, platelet spreading, secretion, and aggregation via the activation of Rac effectors such as WAVE and Arp2/3 complex system or the PAK kinases [93].

### 4.3. Role of Cdc42 in Platelet Function

Cdc42 is highly expressed in platelets (estimated copy number per platelet ~ 27,900), and it is a well-known regulator of filopodia formation in multiple cell types, including platelets [21,73,94,95]. Expression of dominant-negative Cdc42 constructs suppressed filopodia formation in response to bradykinin [94]. It is reported that Cdc42, like Rac1, is translocated to the platelet cytoskeleton upon activation [96]. Activated Cdc42 activates WASP, which recruits and activates the actin-nucleating complex Arp2/3. The Arp2/3 complex nucleates the actin filaments and promotes the growth of new filaments generating a branched actin network [97,98]. Actin cross-linking proteins, like fascin, bundle multiple filaments together, which elongate to form filopodia [99]. Additionally, Cdc42 binds and activates IRSp53 via mDia1, which recruits the Ena/vasodilator-stimulated phosphoprotein (VASP) family protein, Mena, promoting filopodia elongation [100,101].

Experiments using Pf4-Cre Cdc42^fl/fl^ showed that Cdc42 is involved in platelet production since these mice had shortened platelet lifespan and mild macrothrombocytopenia [102]. Surprisingly, they found that Cdc42-/-platelets could form filopodia on fibrinogen-coated surfaces, but the filopodia were defective on vWF suggesting Cdc42 might couple with GPIb. These platelets also had higher secretion and higher aggregation under low agonist concentrations. Finally, they showed that Cdc42-/- platelets formed larger aggregates on collagen under flow and had an accelerated arterial occlusive thrombus formation but prolonged bleeding time [102]. Another study using the conditional knockout Mx-Cre Cdc42^fl/fl^ mice also had macrothrombocytopenia and significantly prolonged bleeding times similar to the Pf4-Cre Cdc42^fl/fl^ mice [21]. This study established a bonafide role of Cdc42in the regulation of platelet activation induced by agonists that activate platelets by GPVI-dependent as well as GPVI –independent signaling [21]. In contrast to the findings of the Pf4-Cre Cdc42^fl/fl^ study, this study showed that Cdc42-/- platelets exhibited diminished phosphorylation of PAK1/2, inhibition of filopodia formation on immobilized CRP or fibrinogen, inhibition of CRP- or thrombin-induced platelet aggregation, secretion of ATP, the release of P-selectin, and minimal phosphorylation of Akt. This study showed that Cdc42 is essential for platelet filopodia formation, secretion, and aggregation [21].

Pharmacological targeting of Cdc42 activity using a Cdc42 activity-specific inhibitor (CASIN), a reversible, small-molecule Cdc42 Activity-Specific Inhibitor, in platelets showed that it selectively inhibited activation of Cdc42 and its downstream effector PAK1/2 [22]. The findings from this inhibitor study align with the Mx-Cre Cdc42^fl/fl^ conditional knockout study since both show that Cdc42 inhibition suppressed platelet aggregation, secretion of ATP, the release of P-selectin, and the phosphorylation of ERK, p38-MAPK, and Akt. Administration of CASIN to mice inhibited collagen-induced platelet aggregation but not the murine tail bleeding time, suggesting that CASIN prevents platelet aggregation without adversely affecting the hemostatic response. CASIN also effectively suppressed laser-induced thrombus formation in vivo, highlighting the antithrombotic effect of Cdc42 inhibition. These studies put Cdc42 as a potential target for developing antithrombotic therapies [22].

### 4.4. RhoGTPases and Signaling Crosstalk in Platelet Function

Platelets utilize dynamic processes involving multiple feedback loops and crosstalks between different regulatory pathways activation [52]. Specifically, platelets depend on the signal amplification mechanisms to regulate cross-talk between phosphorylation-, cyclic nucleotides-, GPCR-, integrins-, RhoGTPases-, calcium-, and lipid-based signaling to regulate platelet function [10,23,52]. RhoGTPases also serve as regulators of ROS by modulating the nicotinamide adenine dinucleotide phosphate (NADPH) oxidase complex. Rac1 binds to p67^phox^ to regulate NADPH oxidase complex assembly, while RhoA regulates ROS formation by inhibiting p47^phox^ phosphorylation [16]. RhoGTPases orchestrate cytoskeletal rearrangement and oxidative stress to generate a positive feedback loop on platelet activation, promoting further ROS production and amplifying platelet response resulting in thrombus formation [103].

Additionally, there is evidence of crosstalk among the RhoGTPases themselves [104,105]. Studies using pharmacologic inhibitors of RhoA, Rac1, and Cdc42 showed that Cdc42 and RhoA regulate platelet aggregation in parallel pathways by possibly affecting the RhoA/ROCK- Mitogen-activated protein kinase (MAPK)-dependent and -independent phosphorylation of MLC [104]. Inhibition of PAK in platelets showed that Rac1 and Cdc42 activation is linked to PAK effector function, which mediates MAPK activation, calcium signaling, and platelet actin dynamics [105]. These studies highlight the crosstalk among RhoA, Rac1, and Cdc42 in various signaling cascades involved in platelet activation.

## 5. Pharmacological Targeting Rho GTPases as an Antiplatelet Approach

**Figure 3:**
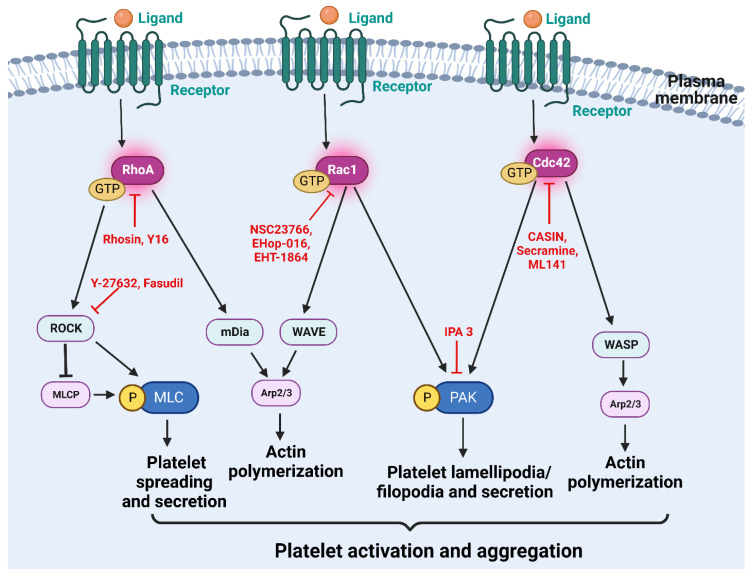
Pharmaceutical targeting of Rho GTPases as an antiplatelet approach. Various pharmacological inhibitors have been developed to selectively target critical components of the Rho GTPase signaling pathways and inhibit platelet activation. RhoA inhibitors such as Rhosin, Y16, and ROCK inhibitors such as Y27632 and Fasudil have been used to study the role of RhoA in platelet function. NSC23766, EHop-016, and EHT-1864 inhibit the activation of Rac1, while CASIN, Secramine, and ML141 inhibit the activation of Cdc42 to study their roles in platelet activation. IPA 3, an inhibitor of PAK, is used to elucidate the role of PAK in platelet function.

Rho GTPases, their upstream regulators, and downstream effectors play a crucial role in platelet activation and aggregation, making them potential targets for developing antiplatelet agents (Figure 3). However, the Rho GTPases have been considered “undruggable” due to efficiency, specificity, and/or druggability limitations. Medicinal chemists initially explored the development of nucleotide analog Rho GTPase inhibitors similar to protein kinases, but Rho GTPases have shown a sub-nanomolar binding affinity for GTP or GDP while the cellular concentration of the GTP was in the micromolar range [106,107]. The surface of the Rho GTPase is relatively smooth apart from the nucleotide-binding pocket since most Ras proteins are mainly involved in protein-protein interactions (PPIs). PPIs are hard to be targeted via small molecule inhibitors due to intractable large shallow surfaces. As mentioned earlier, bacterial toxins target Rho GTPases activity [108]. A well-known bacterial toxin, exoenzyme C3 transferase, an ADP-ribosyltransferase from Clostridium botulinum, has been shown to inactivate RhoA, RhoB, and RhoC but not Rac or Cdc42 [109]. Large clostridial cytotoxins, such as Clostridium difficile toxin A and B glucosylate, Rho GTPases to prevent effector coupling to inhibit Rho functions [110,111]. Although these bacterial toxins have been used to study Rho GTPase function, they have severe limitations, such as the introduction of covalent modifications to the substrates, non-specific interactions, and off-target effects, making them unsuitable for clinical advancement. Other alternative inhibitors targeted the Rho post-translational modifications by interfering with the membrane localization of GTPases. Rho GTPases require proper subcellular localization to function as molecular switches at defined intracellular sites. Rho GTPases translocation to the cell membranes is mediated via post-translational modifications and signaling events that release the Rho-GDP from the GDIs [112]. Inhibitors that attempted to interfere with membrane localization of the GTPases, such as farnesyltransferase inhibitors (FTIs) and geranylgeranyltransferase inhibitors (GGTIs), were pursued but had limitations due to inaccurate preclinical models, off-target effects, and toxicity [113,114].

One of the most popular targeting strategies to inhibit Rho GTPase activation is targeting the RhoGEF-Rho interface since it is essential for catalyzing the GDP-GTP nucleotide exchange of the Rho GTPases. Several small molecule inhibitors that bind to the GEF-binding surface on the Rho GTPase to prevent its activation have been developed. Structure-based rational design strategy coupled with in silico and protein binding screening has led to the identifying a Rho-specific inhibitor, Rhosin (also termed G04). Rhosin specifically binds to RhoA with micromolar affinity to inhibit GEF interaction, RhoA activation, and RhoA-mediated cellular functions [115]. Treating platelets with Rhosin resulted in an inhibition of platelet activation, ROS generation, platelet spreading, secretion, and platelet aggregation, in addition to reduced phosphorylation of MLC [16]. Another targeting strategy is to prevent interaction between specific RhoGEFs and their Rho GTPases. Fluorescence polarization guanine nucleotide-binding assay led to the development of five inhibitors specific to the RhoA-LARG interaction [116]. In separate studies, virtual screening coupled with high throughput screening resulted in the identification of Y16, an inhibitor that binds to the hinge region of the LARG DH/PH domain to inhibit RhoA activation and signaling [117]. The Rho GTPase downstream effectors are classical kinases making them excellent targets for conventional drug design. ROCK inhibitors have been the most promising among all therapeutic interventions of Rho signaling. Fasudil, or HA-1077, an ATP-competitive kinase inhibitor, is the only clinically approved ROCK inhibitor for treating cerebral vasospasms and pulmonary hypertension [118]. Another widely used ROCK inhibitor, Y-27632, is a pyridine-analog that competes with ATP for binding to ROCKs [119]. Platelets treated with Y-27632 inhibited shape change, F-actin polymerization, and phosphorylation of MYPT and MLC [118,120,121].

Structure-based virtual screening of compounds to fit into the surface groove of Rac1 has led to the development of NSC23766 [122]. NSC23766 specifically inhibits Rac1 activation by the Rac-specific GEFs Trio or Tiam1, but not Vav, in a dose-dependent manner. In platelets, Rac1-specific inhibitor NSC23766 blocked dense granule secretion, aggregation, and Rac1 activation [18,123]. Additionally, small molecule targeting of Rac1-p67^phox^ interaction by Phox-I prevented GPVI- and non-GPVI-mediated NOX2 activation, ROS generation, and platelet function without affecting the bleeding time [91]. Medicinal chemists identified, synthesized, and characterized the biological activity of EHop-016, a novel NSC23766 derivative that is 100-fold more effective at inhibiting Rac1 activation [124]. Both NSC23766 and EHop-016 can reduce Rac-directed lamellipodia formation and the activity of Rac downstream effector PAK. Another small molecule Rac inhibitor, EHT 1864, inhibits Rac downstream signaling and transformation by a novel mechanism involving guanine nucleotide displacement [125].

CASIN inhibits Cdc42 activity by blocking the GEF interaction with submicromolar affinity, resulting in specific, reversible, and dose-dependent inhibition [126,127]. CASIN blocked platelet filopodia formation, secretion, and platelet aggregation along with the phosphorylation of its downstream effector PAK. CASIN treatment resulted in the formation of small and unstable thrombi. CASIN also inhibited ex vivo aggregation but did not affect the murine tail bleeding times [22]. High-throughput synthesis and phenotypic screening led to the discovery of Secramine, a molecule that inhibits membrane traffic via RhoGDI. It is reported that Secramine specifically inhibits the activation of Cdc42 antagonizing its membrane association, nucleotide exchange, and effector binding in a RhoGDI-dependent manner [128]. Secramine blocked Cdc42 activation, platelet adhesion, filopodia formation, and aggregation [20,129]. High-throughput screening and decreased fluorescent GTP binding were used to identify ML141 or CID29950007, a novel Cdc42 inhibitor [130]. This compound blocks the nucleotide-binding independent of the effects of GEF activity and is a potent, selective, and reversible non-competitive inhibitor of Cdc42 activity [131]. PAK inhibitor, IPA3, targets the autoregulatory mechanism and promotes the inactive conformation of PAKs. IPA 3 is an allosteric inhibitor that covalently binds to PAK proteins inhibiting the action of PAK by its RhoGTPases Cdc42 and Rac [132,133]. Studies using IPA 3 showed that inhibition of PAK prevented thrombin-induced platelet aggregation in addition to blocked platelet focal adhesion and lamellipodia formation [105].

These inhibitor studies indicate that pharmacologic targeting of Rho GTPases by specific and reversible inhibitors makes it possible to better define their involvement in homeostatic modulation and leads to the discovery of novel antiplatelet agents.

## 6. Future Perspectives and Conclusions

Platelets have been valuable tools and have been a necessary cell type in studying the signal transduction mechanisms of Rho GTPases over the past two decades [10,23,25,70]. The studies highlighted here helped identify the crucial roles of RhoA, Rac1, and Cdc42 in platelet function and in mediating the thrombosis and hemostasis processes. There is growing evidence that these Rho GTPases are a part of more complex signaling networks, with a significant amount of overlap and crosstalk involving their upstream regulators and downstream effectors in regulating platelet hemostasis and thrombosis. With the development of better genetic models, technological improvements in cell biology and pathophysiology, and the advancement of Rho GTPase-specific pharmacological targeting, there is high hope that further studying Rho GTPase intervention will yield promises for future therapeutics of platelet-mediated diseases.
